# Hybrid robot & FES rehabilitation after stroke: Short‐term performance, fatigue, and patient experience

**DOI:** 10.1177/20556683261488467

**Published:** 2026-09-23

**Authors:** Lucille Cazenave, Chiara Höhler, Satoshi Endo, Hossein Kavianirad, Nuria Peña-Pérez, Lisa-Marie Huber, Aaron Yurkewich, Thierry Keller, Sandra Hirche, Klaus Jahn, Etienne Burdet, Carmen Krewer

**Affiliations:** 1 AI for Healthcare Centre for Doctoral Training, 4615 Imperial College of Science, Technology and Medicine, London, UK; 2 Bioengineering Department, 4615 Imperial College of Science, Technology and Medicine, London, UK; 3 Research Department of Schoen Clinic Bad Aibling, Bad Aibling, Germany; 4 Chair of Information-oriented Control, Technical University of Munich, Germany; 5 Institute for Automatic Control, 9165 RWTH, Aachen, Germany; 6 Department of Mechatronics Engineering, 85458 Ontario TechUniversity, Oshawa, Canada; 7 152686 Tecnalia Research and Innovation, San Sebastian, Spain; 8 International Consortium for Rehabilitation Technology, Zurich, Switzerland

**Keywords:** Neurorehabilitation, Stroke Rehabilitation, Robot-assisted rehabilitation, Electrical Stimulation, Rehabilitation Devices

## Abstract

Combining functional electrical stimulation (FES) and robotics may enhance post-stroke motor recovery, particularly in the subacute phase, yet evidence on usability and user experience remains limited. This study evaluated the feasibility and short-term effects of robotic, FES, and hybrid assistance during a wrist flexion–extension tracking task in 32 subacute stroke patients, most with severe upper-limb impairments. Participants received one of the three assistance modalities across three training sessions. Quantitative measures of tracking performance, effort, and fatigue, along with patient-reported outcomes, were analysed. Tracking performance improved during assisted trials across all groups, confirming the immediate efficacy of each modality, with no significant differences between them. The benefit of assistance was greater in more impaired patients, particularly in the Robot and Hybrid groups. Cocontraction increased in the FES and Hybrid groups during assistance, suggesting enhanced muscular engagement, while no change was observed in the Robot group. No significant increases in muscular fatigue were detected. All modalities were well tolerated, enjoyable and perceived as useful, with hybrid assistance receiving consistently positive feedback. Although the short intervention limited conclusions on unassisted performance over time, these findings demonstrate the feasibility and acceptability of hybrid robot–FES training across a broad range of impairment levels.

## Introduction

Sensorimotor rehabilitation is essential after stroke to reduce disability, yet current services often fall short of patients’ expectations, particularly regarding upper-limb recovery.^1,2^ While the recovery outcome depends on the lesion, numerous technological approaches have been developed to complement conventional therapy, and provide high-intensity, repetitive training, that could enhance recovery. Robotic systems enable precise and consistent guidance of movements,^3^ while FES can directly activate weak muscles and enhances sensorimotor engagement.^4^ Although both technologies have shown benefits in post-stroke rehabilitation, most clinical evidence has been obtained in chronic or moderately impaired populations. In a meta-analysis of robot-assisted therapy, only 10 of 38 randomised controlled trials (RCTs) included patients early post-stroke,^5^ and in a systematic review of FES therapy, only 5 of 18 studies involved participants less than three months post-stroke.^6^

Additionally, both modalities have limitations: robotic-only therapy may reduce active patient participation,^7–10^ whereas FES-only therapy can be limited by rapid onset of muscle fatigue and suboptimal motor unit recruitment patterns, the presence of high tone or spasticity or abnormal limb posture during stimulation.^11^ These complementary strengths and weaknesses have motivated the development of hybrid rehabilitation systems combining robotic and FES assistance.

Several clinical studies have reported superior functional outcomes with hybrid systems compared to conventional or robot-only therapy, including improvements in Box and Block and Wolf Motor Function Test performance.^12–15^ However, most of these studies have focused on technical performance or motor outcomes, rather than on the feasibility, tolerability, and patient acceptance of hybrid systems in clinical populations.^16^ Additionally, a recent systematic review with meta-analysis evaluated the efficacy of hybrid neuroprostheses for upper limb rehabilitation after stroke.^17^ It was found that such intervention has a positive effect on upper limb recovery after stroke both in the sub-acute and chronic stage, with effects persisting at least three months after the intervention. However, many existing systems have implemented only partial combinations of the two modalities, for example using robotics passively or relying on manual triggering of stimulation.^16^ More specifically, half of the hybrid systems from the systematic review^17^ used an active robotic system applied to the same joint as the FES. Such designs may limit the synergistic potential of hybrid rehabilitation, as patient engagement and active contribution are key determinants of neuroplasticity and recovery.^18^ Moreover, only 3 out of the 7 RCTs identified were conducted in subacute stroke patients and 3 included participants with severe upper-limb impairments in the subacute stage of stroke.

These observations indicate that the feasibility and tolerability of these technologies have been comparatively less investigated in subacute and more severely impaired patient groups, although early initiation of motor rehabilitation is known to promote better functional outcomes after stroke.^19^ The present work addresses this gap by evaluating the feasibility of a hybrid system that combines robotic support and FES through an impedance controller, applied to wrist flexion–extension during a continuous target-tracking task (Figure 1A). This system and task have previously been validated in unimpaired participants, demonstrating improved performance, and increased voluntary engagement without inducing fatigue.^20^ Here, we carry out these investigations with stroke patients to assess the translational potential of the hybrid approach in a clinical setting. This work extends on preliminary results from,^21^ by including the full cohort of patients across all three intervention groups and providing a more comprehensive evaluation, including detailed statistical analyses. Collecting both quantitative performance and physiological measures and qualitative user feedback in stroke patients provides further evidence on the combined use of robotic and FES assistance at the same joint. Specifically, we aimed to (i) examine user experience (e.g., patients’ subjective perceptions of comfort, enjoyment, usefulness, and assistance) (ii) compare the effects of robotic-only, FES-only, and combined hybrid assistance on task performance, effort, and fatigue, and (iii) evaluate short-term training effects on unassisted performance across repeated sessions. By combining behavioural, physiological, and user-centred outcomes, this study provides a comprehensive evaluation of the potential of hybrid assistance for upper-limb rehabilitation post-stroke.

**Figure 1.**
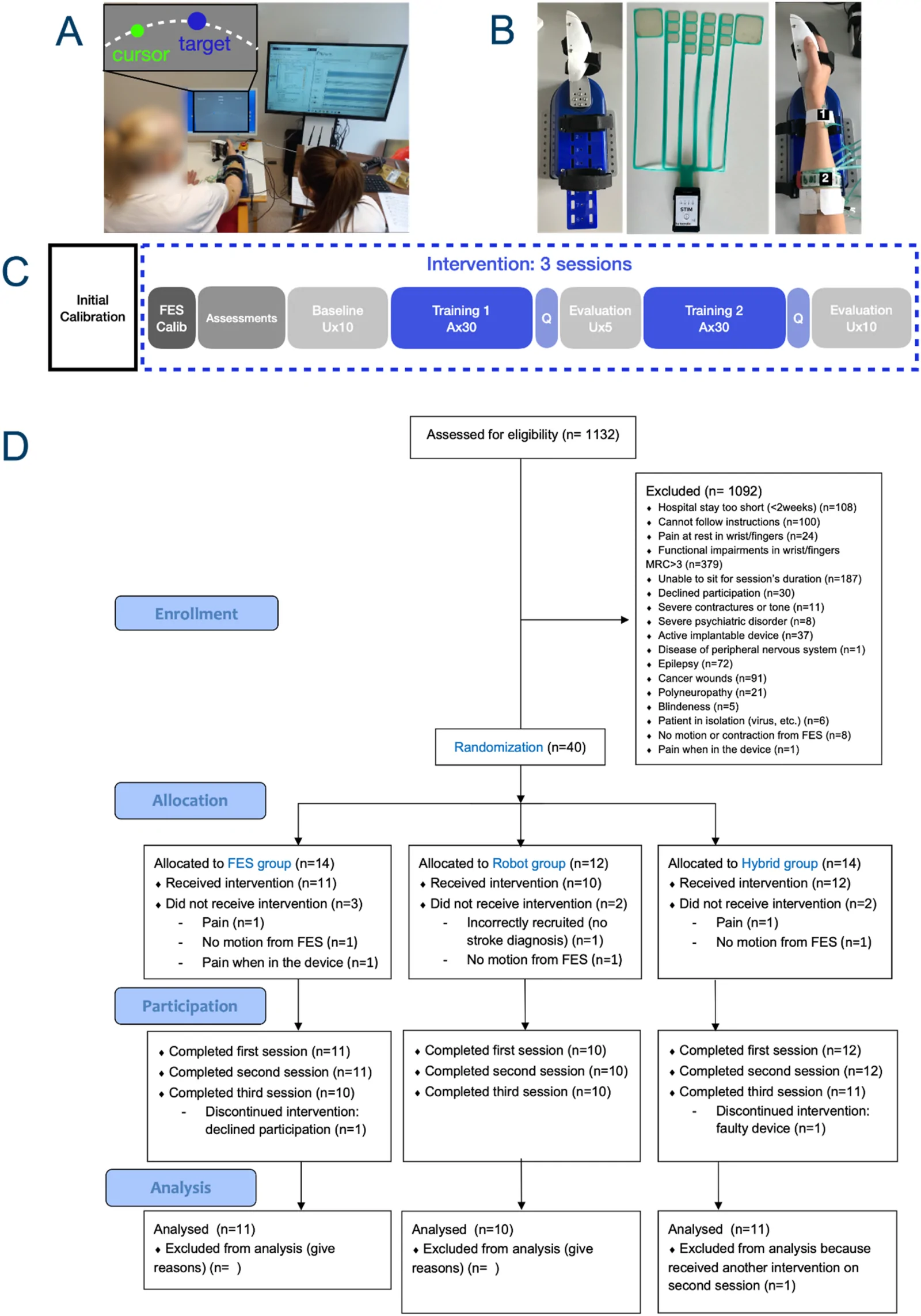
Experimental protocol and setup. A. A participant performs a wrist flexion–extension task by tracking a randomly moving on-screen target. B. Hybrid system components (from left to right): HRX-1 wrist robotic device; FES system equipped with a 16-electrode array; and the combined hybrid configuration. In this setup, the FES anode electrodes (labelled “1”) are positioned on the dorsal and ventral aspects of the wrist near the ulnar head, while the cathode electrode array (labelled “2”) is aligned with the wrist flexor and extensor muscle bellies. The bipolar EMG electrodes were placed on the wrist muscles between the anode and the cathode. C. Schematic of the experimental protocol. Participants complete an initial sequence of calibration and assessment steps, followed by unassisted trials (U), during which no robotic or electrical assistance is applied, and assisted training trials (A). The first assisted block and evaluation block are followed by a questionnaire (“Q”). “×10” indicates the number of repetitions per block (ten trials). D. Consolidated Standards of Reporting Trials (CONSORT) diagram, showing participants screening, group allocation, participation and data analysis.

## Methods

### Participants

The study was run at the Schön Klinik Bad Aibling and was approved by the Ethics Committee of the Ludwig-Maximilians University (LMU) Munich, Germany (number: 22-0297, DRKS registration: 00030586). All participants or their legal representatives signed an informed consent form prior to participating in the experiment. Participants were subacute stroke patients admitted to an inpatient rehabilitation hospital following either an ischaemic or haemorrhagic stroke.

Demographic information was extracted from medical records, and motor impairment severity at baseline was assessed using the Fugl–Meyer Upper Extremity (FM-UE) scale.

Eligibility criteria included: age above 18 years, the cognitive ability to understand and follow instructions, and the presence of functional impairments affecting the wrist or fingers, defined by the UKMedical Research Council (MRC) score of 3 or below. In addition, only participants reporting minimal or no pain in the wrist or fingers, corresponding to a Numeric Rating Scale (NRS) score below 4 were considered for inclusion.

Exclusion criteria were defined separately for the robotic device and the FES intervention. For the robotic component, patients were excluded if severe contractures or increased muscle tone prevented correct positioning of the arm within the device or limited wrist flexion–extension to less than 20°. For the FES component, exclusion criteria included pregnancy, the presence of implantable electronic devices or metallic implants in the stimulation area, absence of visible muscle contraction or movement in response to stimulation, severe epilepsy, cancer, or open wounds in the targeted region.

Finally, patients were excluded in the presence of severe psychiatric disorders, or if both motor function and deep sensory function in the wrist or fingers were absent.

### Hybrid Robot-FES system

The Hybrid Robot-FES system used in this study (Figure 1B) is composed of a one degree-of-freedom wrist flexion/extension robotic interface (HumanRobotiX, UK)^22^ (Figure 1B, left) and an FES device (Tecnalia Research & Innovation, Spain) (Figure 1B, middle) providing stimulation to the wrist flexor/extensor muscles. It has been described and tested in our previous work in unimpaired participants^20^ and stroke patients.^21^

Muscle activity of the wrist flexor (flexor carpi radialis) and wrist extensor (extensor carpi radialis longus) is recorded at a frequency of 1500 Hz using bipolar EMG electrodes (Noraxon, USA).

### Protocol

The overall experimental protocol is illustrated in (Figure 1C). Following enrolment, participants were randomly allocated to one of three intervention groups: Robot, FES, or Hybrid (Robot+FES), which defined the type of assistance delivered during the training phase across the three sessions. Before the intervention phase, an initial calibration session was conducted to familiarise participants with the system and to perform the first FES calibration. Subsequently, each participant completed three intervention sessions of approximately one hour, scheduled with at least one rest day between sessions. All intervention sessions followed the same structure, beginning with FES calibration. During this step, electrode placement was adjusted to ensure both comfort and effective stimulation, and individual stimulation parameters were determined, including the sensory threshold and the maximum level of comfortable stimulation. It is followed by a passive and active range of motion (ROM) calibrations. Further details about these procedures can be found in our previous work.^20,21^

### Experimental task

During the remainder of each session, participants performed a wrist flexion-extension target-tracking task using their impaired hand. The angular position of the participant’s wrist, measured by the robot encoder, was linearly mapped to the on-screen cursor position, while the target cursor followed a predefined trajectory. Participants were instructed to voluntarily follow the target as accurately as possible by flexing and extending their wrist. Thus, tracking performance was based solely on wrist angle and was independent of the EMG recordings, which were acquired only for subsequent analysis of muscle activity.

Trials were conducted either without assistance (baseline and evaluation trials in Figure 1C), where no FES or robotic support was applied, or with assistance (training blocks), during which participants received FES and/or robotic assistance depending on their assigned group. Each session began with ten unassisted baseline trials. The first training block comprised 30 assisted trials, followed by five unassisted trials for intermediate evaluation. If time permitted and participants remained comfortable and attentive, a second training block of 30 assisted trials was performed, followed by a final evaluation block of ten unassisted trials. If the second training block was not completed, five additional unassisted trials were added so that the session consistently ended with ten unassisted evaluation trials.

Each trial lasted 12 seconds and was followed by a 3-second break, which could be extended at the participant’s request. During each trial, participants tracked a moving target displayed on a monitor positioned in front of them (Figure 1A), with instructions to follow it as accurately as possible. The definition of the target trajectory can be found in our previous work.^21^

### Questionnaire

Following the first training block and subsequent evaluation block, participants completed a questionnaire assessing perceived workload, using the NASA-TLX ten-point ordinal Likert Scale.^23^ They also responded to their agreements to various statements, using five-point ordinal Likert Scale,^24^ about the comfort of the system (‘The device was comfortable’), the perception of the assistance (‘During the experiment I felt some assistance’), and the usefulness of the assistance (‘The device improved my performance’), and to two statements derived from the enjoyment subscale of the Intrinsic Motivation Inventory^25^: ‘I enjoyed this activity very much’ and ‘I would like to use this device in my therapy’.

### Hybrid Robot-FES controller

The hybrid controller has been described in detail in our previous work.^20^ Briefly, assistance was derived from the participant’s tracking error, defined as the angular difference between the participant’s wrist position and the target trajectory. The controller applied assistance proportional to this error using an impedance-based control law. Consequently, larger tracking errors resulted in larger assistive torques and/or stimulation amplitudes.

Depending on the intervention group, assistance was delivered through the robot, FES, or both. In the Robot group, assistance was provided exclusively through robotic torque. In the FES group, assistance was delivered exclusively through electrical stimulation of the wrist flexor or extensor muscles. In the Hybrid group, patients received the same robotic assistance as those in the Robot group together with the same FES assistance as those in the FES group, applied simultaneously. Voluntary EMG activity was not used to control the assistance but was recorded solely for physiological analyses.

### Data analysis

In this work, we collected both qualitative and quantitative data to to compare the effects of robotic assistance, FES, and their combined use on patients’ perceptions of the system’s comfort, the usefulness of the assistance and their enjoyment of the task, as well as to measure performance, effort and fatigue changes in the task under the three assistance modalities and over three sessions. We investigated patient experience of the assistive modalities by analysing the average questionnaire answers across the three sessions (for the unassisted and assisted blocks separately). We also explored the effect of the three assistance modalities on patients’ performance, effort, and fatigue, as well as the effect of training with the system over three sessions on these metrics.

Performance was measured using the *root mean square error* (RMSE) in degrees:

(6) $${\left(\frac{1}{T}\;\overset{T}{\underset{0}{\int }}{e\left(t\right)}^{2}\;dt\;\right)}^{1/2}$$

with T the trial duration. RMSE was normalised by the target’s range as this was calibrated to each patient’s ROM. Additionally, the Spearman’s linear *correlation between the frequency spectra* of the target trajectory and that of the participant was also used to quantify how well participants followed the target as well as whether their movement was jerky, i.e., containing high-frequency components.

Muscle activity was characterised using *cocontraction* (CC) and *reciprocal activation* (RA) indices derived from the envelope of the pre-processed surface EMG recordings, when these were available (EMG was not recorded for ten patient-session pairs due to technical issues). EMG pre-processing consisted of Notch filtering at the mains noise frequency of 50 Hz, followed by high-pass and low-pass second order Butterworth filters (with cut-off frequencies *f*_*c*_ = 10 Hz and *f*_*c*_ = 500 Hz, respectively).

Additionally, a blanking filter with a window of 10 ms was used to remove the FES artifacts and an adaptive filter designed for EMG-based torque estimation under stimulation of wrist flexor and extensor with the same setup as here^26^ was used to further remove the FES-induced M-wave components. To obtain the envelope of the EMG, the filtered EMG was rectified and filtered with a low-pass second order Butterworth filter with cut-off frequency *fc* = 5 Hz. The EMG envelope was then normalised by the maximum contraction obtained during the EMG calibration phase, where patients had to resist various force levels in the flexion and extension directions. Cocontraction (CC) and reciprocal activation (RA) were computed from this filtered EMG:

(7) $$CC=min\left(EMG_{flex},EMG_{ext}\right),$$

$$RA=\max\left({EMG}_{flex},{EMG}_{ext}\right)-CC.$$

Fatigue metrics were computed and investigated as indicated in Supplemental File 2.

All metrics were calculated for each trial during the ten baseline trials and the 30 trials of the first training block, which are the two phases of the experiment (unassisted, assisted). Mean values were computed across baseline and training trials and used for subsequent analysis.

### Statistical analysis

For the questionnaire answers, Kruskal-Wallis rank sum test (between-subject: intervention group) was used to investigate a potential effect of the intervention group, due to ordinal data and non-normal distributions.

To assess the effects of assistance, training, and impairment on task performance and muscle activation, linear mixed-effects models (LMEs) with restricted maximum likelihood estimation were fitted. The dependent variables were the four primary outcomes: normalised RMSE, spectral correlation, normalised cocontraction, and normalised reciprocal activation.

Fixed effects included *Phase* (unassisted U1 vs. assisted A1), *Session* (numeric predictor for sessions 1, 2, 3), *Group* (FES, Robot, Hybrid), and baseline motor impairment *FM-UE* (centered FM-UE score). The Phase factor was considered to assess whether the assistance provided to patients during the target-tracking task was related to a performance increase, and whether differences were observed in the extent to which these assistive modalities can help. The Session factor was used to capture changes over the three training sessions. Two-way interactions between FM-UE and Phase or Session were included to test whether the assistance effects or the metrics trends over time changed with patients’ the impairment level. Patients’ IDs were included as a random intercept to account for repeated measures within participants.

Omnibus significance of fixed effects was evaluated with Type III F-tests using Satterthwaite’s approximation for degrees of freedom.

When main effects or interactions were significant, planned comparisons were conducted with estimated marginal means: (i) Phase contrasts (assisted A1 vs. unassisted U1) globally and/or within Groups, (ii) Session slopes (training trends) within Groups and/or Phases, (iii) Comparisons of FM-UE slopes between the two Phases. P-values for multiple contrasts were adjusted with Bonferroni-Holm’s method.

All analyses followed an intention-to-treat principle, whereby all available data were included even when some sessions were missing, except for longitudinal (3 x 3) analyses, which required complete data across all sessions. A significance level of 0.05 was applied to all statistical tests.

## Results

### Participants

1132 patients were screened from October 25^th^, 2022, to June 6^th^, 2025 (Figure 1D). 40 patients were recruited and 30 completed the full three-session intervention (Table 1). 32 patients completed the first two sessions and were included in the analyses of the assistive effect and the patient experience, while only patients who completed all three sessions were included in the analyses of the training effect. Twenty-two patients presented with severe impairment (Fugl–Meyer Upper Extremity score, FM-UE ≤ 25), four with moderate impairment (25 < FM-UE ≤ 40), and four with mild impairment (FM-UE > 40). The FM-UE score is missing for two patients out of the 32 patients who were considered for analyses. Additionally, the FM-UE baseline scores did not differ significantly across intervention groups (*F*(2,27) = 0.60, *p* = 0.556), indicating that the baseline impairment level of participants was similar across groups. The time since stroke was also not significantly different across groups (*χ*^2^(2) = 4.83, p = 0.089).

**Table 1.** Patient Demographics.

Group	Sex (M | F)	Age, years, mean (sd)	Paretic side (R | L)	Time since stroke, weeks, mean (sd)	FM-UE (/66) mean (sd)
Total	22 | 10	66 (14)	15 | 17	11 (7)	19 (14)
FES	9 | 2	61 (18)	5 | 6	15 (8)	15 (11)
Robot	7 | 3	69 (11)	7 | 3	9 (6)	23 (15)
Hybrid	6 | 5	68 (11)	3 | 8	9 (4)	10 (17)

FM-UE: Fugl-Meyer Upper-Extremity Assessment.

### Patient experience

Regarding the reported comfort of the system, the Kruskal-Wallis tests found no significant group differences (χ^2^(2) = 0.36698, *p* = 0.8324), enjoyment (*χ*^2^(2) = 2.3377, *p* = 0.3107), or desire of use (*χ*^2^(2) = 0.4166, *p* = 0.812) (Figure 2A). Across all metrics, ratings were generally positive (comfort: 1.49 ± 0.58; enjoyment: 1.29 ± 0.83; desire of use: 1.37 ± 0.75), indicating a positive reception of the training regardless of the intervention modality. Comfort ratings were consistently positive across participants, suggesting that none of the assistance modes introduced notable physical discomfort, even when electrical stimulation was applied (FES: 1.42 ± 0.60; Robot: 1.49 ± 0.69; Hybrid: 1.57 ± 0.48). Enjoyment scores were high as well, reflecting good engagement with the task (FES: 1.03 ± 0.97; Robot: 1.20 ± 0.91; Hybrid: 1.62 ± 0.52), while desire of use ratings indicated a general willingness to continue using the device, regardless of modality (FES: 1.22 ± 1.01; Robot: 1.40 ± 0.66; Hybrid: 1.51 ± 0.53). Although the statistics did not support significant between-group effects, the distribution of the data indicated slightly more clustered high ratings in the Hybrid group for the answers regarding patients’ enjoyment and desire to further use the device, potentially due to the perceived benefit of dual-modal assistance or the generally higher assistance level effect (Figure 2A).

**Figure 2.**
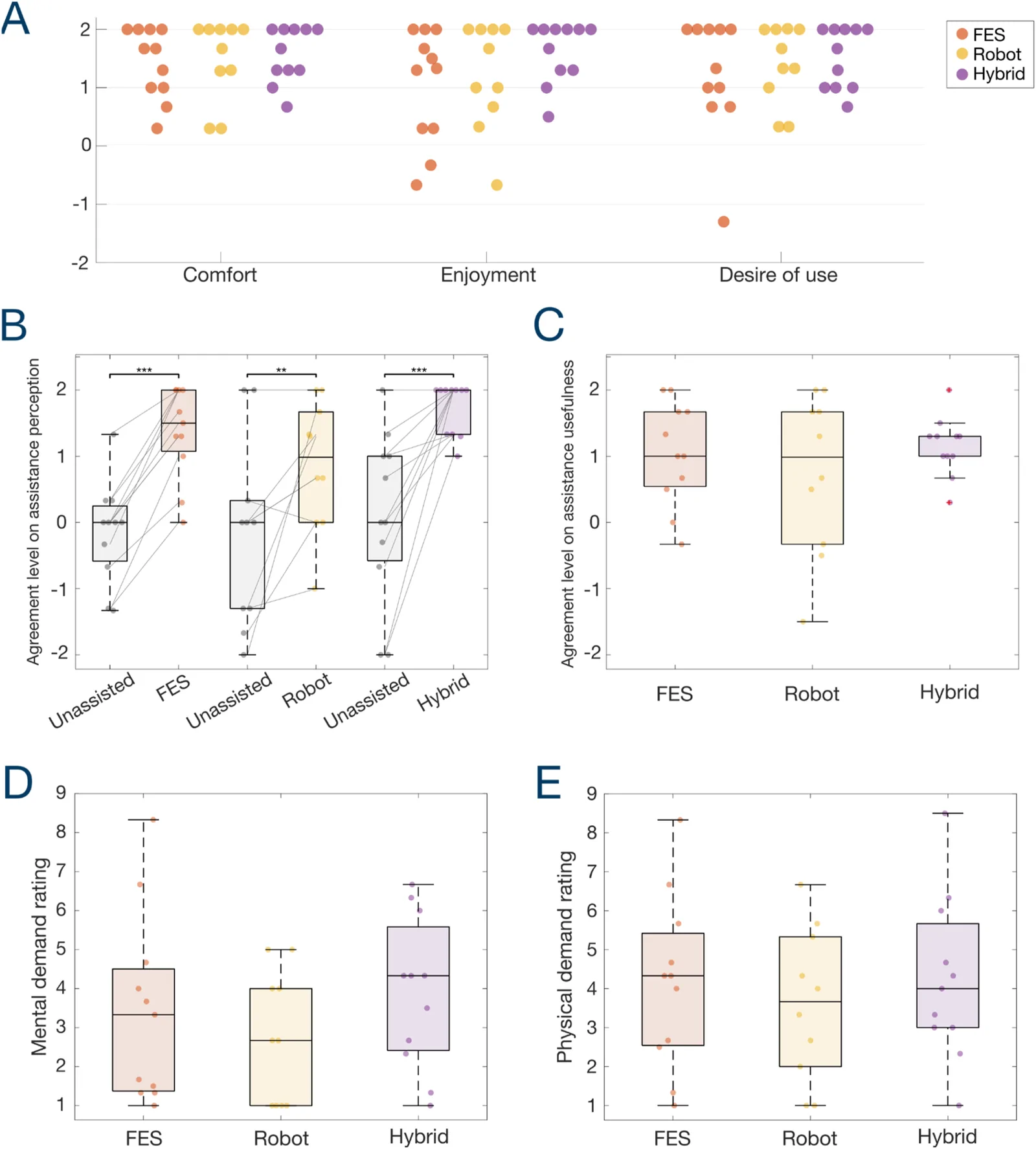
Subjective perception of the unassisted and assisted target-tracking task for each intervention group, reported by patients using a 5-point Likert scale ranging from “strongly disagree” (−2) to “strongly agree” (+2) (A-C) and to the 10-point NASA-TLX questionnaire on perceived effort with answers from very low (1) to very high (10) (D,E). (A) Answers to the questions (figure from left to right): ‘The use of the device was comfortable’, ‘I enjoyed doing this activity very much’, ‘I would like to use this system for my upper-limb therapy in the clinic’. (B) Answers to the question ‘During the experiment, I felt assistance’ after the unassisted and assisted trial. The grey lines are used to connect data points from the same patient under different conditions. (C) Answers to the question ‘The use of the device improved my performance’. (D). Answer to the question: ‘How mentally demanding was this task’. E. Answer to the question: ‘How physically demanding was this task’.

We also investigated whether patients were aware of the provided assistance and their *perception of the assistance* both after the assisted trials and after the unassisted trials (Figure 2B). The 2 × 3 mixed ANOVA (Phase vs Group) indicated a significant main effect of Phase (*F*(1,29) = 56.2901, *p* < 0.0001), but no significant main effect of Group (*F*(2,29) = 1.1410, *p* = 0.3334) or interaction (*F*(2,29) = 0.9664, *p* = 0.3923). Post-hoc comparisons indeed revealed that patients perceived the increase in assistance between the unassisted and assisted trials in all group (FES: *p* = 0.0001; Robot: *p* = 0.004; Hybrid: *p* < 0.0001). Despite the lack of effect of group, patients generally rated their perception of the assistance higher when in the FES and Hybrid groups (FES: 1.37 ± 0.70; Robot: 0.86 ± 0.98; Hybrid: 1.72 ± 0.39), possibly reflecting the more tangible or noticeable nature of electrical stimulation compared to purely mechanical guidance, especially with this compliant controller, but also potentially reflecting the ceiling effect of the questionnaire metric.

Furthermore, we asked patients after the assisted training block how *useful* the assistance was (Figure 2C). Despite answers from patients in the Hybrid group (1.15 ± 0.44) were higher and with less variance than in the FES group (1.05 ± 0.78) and the robot group (0.75 ± 1.20), the Kruskal-Wallis test did not reveal a significant effect of group (FES, Robot, Hybrid) on perceived usefulness of the assistance (*χ*^2^(2) = 2.237, *p* = 0.3268).

Regarding the NASA-TLX answers to the mental and physical demands items, we did not find a significant effect of the intervention group (Robot, FES, Robot+FES) (Figure 2D and E) (mental demand: *χ*^2^(2) = 1.9409, *p* = 0.3789, physical demand: *F*(2,29) = 0.269, *p* = 0.766). The median ratings for both items in all groups were lower than 5/10, indicating a generally low perceived effort for the training task.

### Assistance

We report the effect of assistance (Phase) from the robot, FES, or both, on the performance and EMG metrics. Plots of the mean metric values across sessions in the unassisted and assisted trials for each group are displayed in Figure 3.

**Figure 3.**
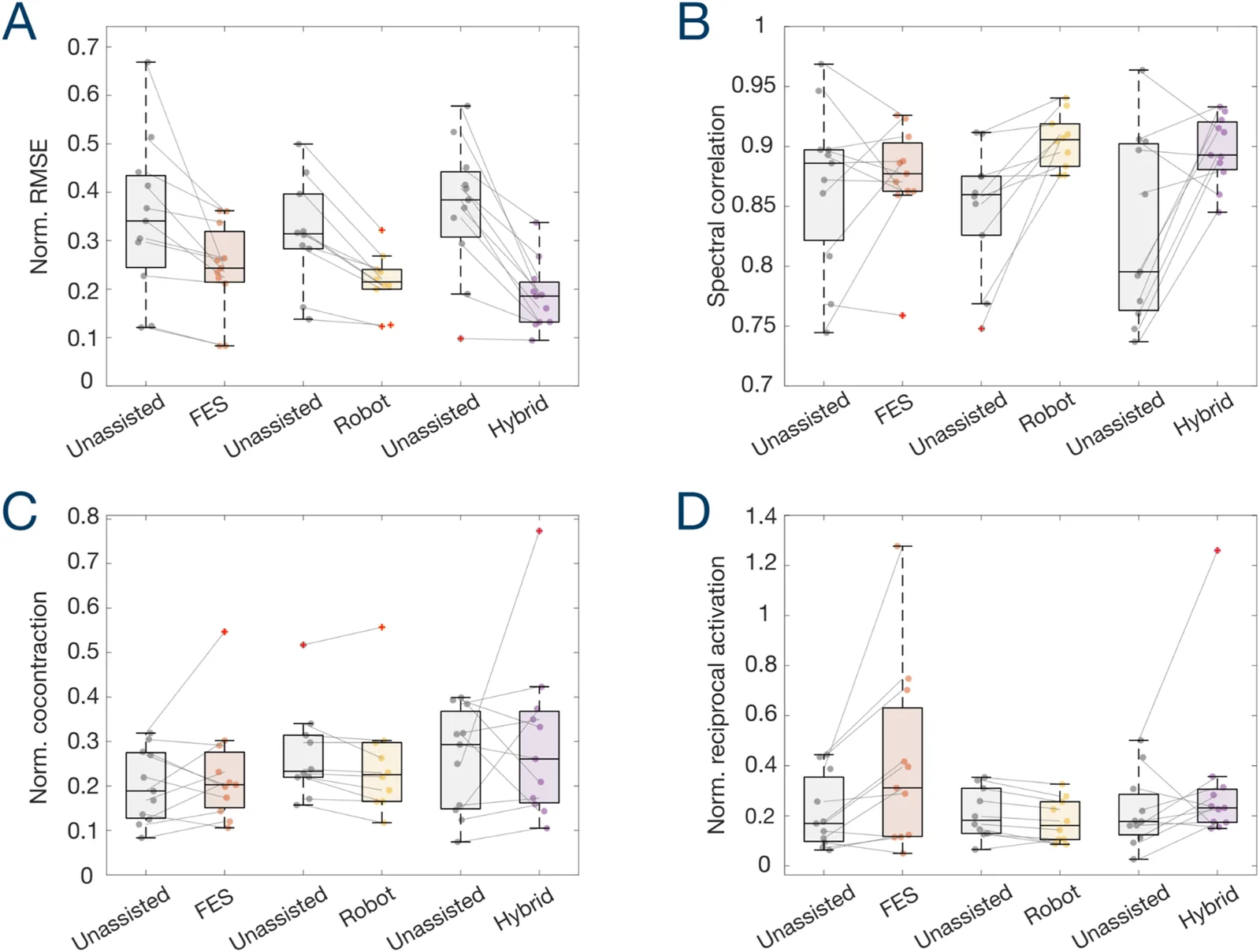
Assistive effect of the FES, robot and hybrid systems during the target-tracking task. The grey lines are used to connect data points from the same patient under different conditions. (A) Effect of the assistance on the normalised RMSE between the patient and target trajectory. (B) Effect of the assistance on the correlation between the frequency spectra of the patient and target trajectory. (C) Effect of the assistance on the cocontraction of the wrist flexor and extensor muscles. (D) Effect of the assistance on the reciprocal activation of the wrist flexor and extensor muscles.

The LME revealed a strong main effect of Phase (*F*(1,132.8) = 13.70, *p* < 0.001) on the normalised RMSE between the patient’s and target trajectories (Figure 3A). A significantly lower RMSE was observed during assisted trials (A1) compared to unassisted trials (U1) (U1–A1 slope difference = 0.134 ± 0.013, *t*(133) = 10.35, *p* < 0.0001). This indicates, as expected, that providing patients with assistance to perform the task increases their performance. However, the assistance modality does not affect this improvement (Group effect: *F*(1,81.4) = 0.4392, *p* = 0.6460). Additionally, a significant Phase × FM-UE interaction was also observed (*F*(1,132.8) = 9.24, *p* = 0.0028), where pairwise contrasts indicated that the reduction in RMSE with assistance was larger in more impaired patients (Supplemental File 1, Figure 6A). The FM-UE slope differed significantly between U1 and A1 (U1–A1 slope difference = −0.00295 ± 0.00097, *t*(133) = −3.04, *p* = 0.0028). This indicates that the benefit of assistance is especially strong for patients with lower FM-UE, who typically cannot perform very well in the unassisted trials due to the lack of volitional movement. No main effect of Group or other interactions reached significance (*p* > 0.06).

For the correlation between the frequency spectra of the patient’s and the target trajectories, the main effect of Phase was not significant (*F*(1,132.1) = 3.33, *p* = 0.070) (Figure 3B), but a Phase × FM-UE interaction was detected (*F*(1,132.1) = 19.64, *p* < 0.0001). Follow-up contrasts confirmed that FM-UE strongly predicted performance improvement with assistance (U1–A1 slope difference = 0.00254 ± 0.00057, *t*(132) = 4.43, *p* < 0.0001). Similarly as with the normalised RMSE, the patients with higher impairment gained proportionally more from the assistance in terms of spectral correlation (Supplemental File 1, Figure 6B). No other main effects or interactions were significant (*p* > 0.07).

For the cocontraction, no main effects of Phase, Group, or FM-UE were observed (all *p* > 0.11) but a three-way Phase × Session × Group interaction (*F*(2,116.78) = 3.23, *p* = 0.043) was found. The cocontraction levels in the unassisted and assisted trials in each group can be seen in Figure 3C. Follow-up analyses revealed group-specific patterns. When comparing assisted and unassisted trials within each group across sessions, patients in the FES group showed significantly lower cocontraction in unassisted than assisted trials (U1–A1 slope difference =– 0.182 ± 0.064, *t*(117) =– 2.85, *p* = 0.005). By contrast, no such Phase differences were found in the Robot group (*p* = 0.88), and patients in the Hybrid group showed a trend towards increased cocontraction with assistance (U1–A1 slope difference =– 0.135 ± 0.068, *t*(117) =– 1.97, *p* = 0.051). Additionally, a significant Phase × FM-UE interaction was observed (*F*(1,116.8) = 6.85, *p* = 0.01003). Planned comparisons showed that FM-UE U1–A1 slopes differed between phases (U1–A1 slope difference = 0.00704 ± 0.00269, *t*(117) = 2.617, *p* = 0.0100), indicating that impairment level modulated the degree to which assistance altered antagonist muscle co-activation. Patients with higher FM-UE tended to show larger reductions in cocontraction in the assisted phase (Supplemental File 1, Figure 6C).

For reciprocal activation (RA), the model revealed no main effect of Phase (*F*(1,115.82) = 0.07, *p* = 0.793) or Group (*F*(2,115.25) = 2.68, *p* = 0.073) (Figure 3D). There was a significant main effect of FM-UE (*F*(1,115.63) = 8.00, *p* = 0.006), and this effect interacted with Phase (Phase × FM-UE, *F*(1,115.82) = 7.20, *p* = 0.008). However, follow-up analyses did not reveal a significant increase in RA with higher FM-UE when investigating each phase separately (*p* > 0.19) (Supplemental File 1, Figure 6D).

### Training

With the LMEs, we also investigated the temporal effect of training with the robot, FES or both over three sessions and examined any potential changes, specifically in the unassisted performance and muscle activity metrics of patients, which may indicate some task learning. Plots of the metric values over sessions in the unassisted and assisted trials for each group are displayed in Figure 4.

**Figure 4.**
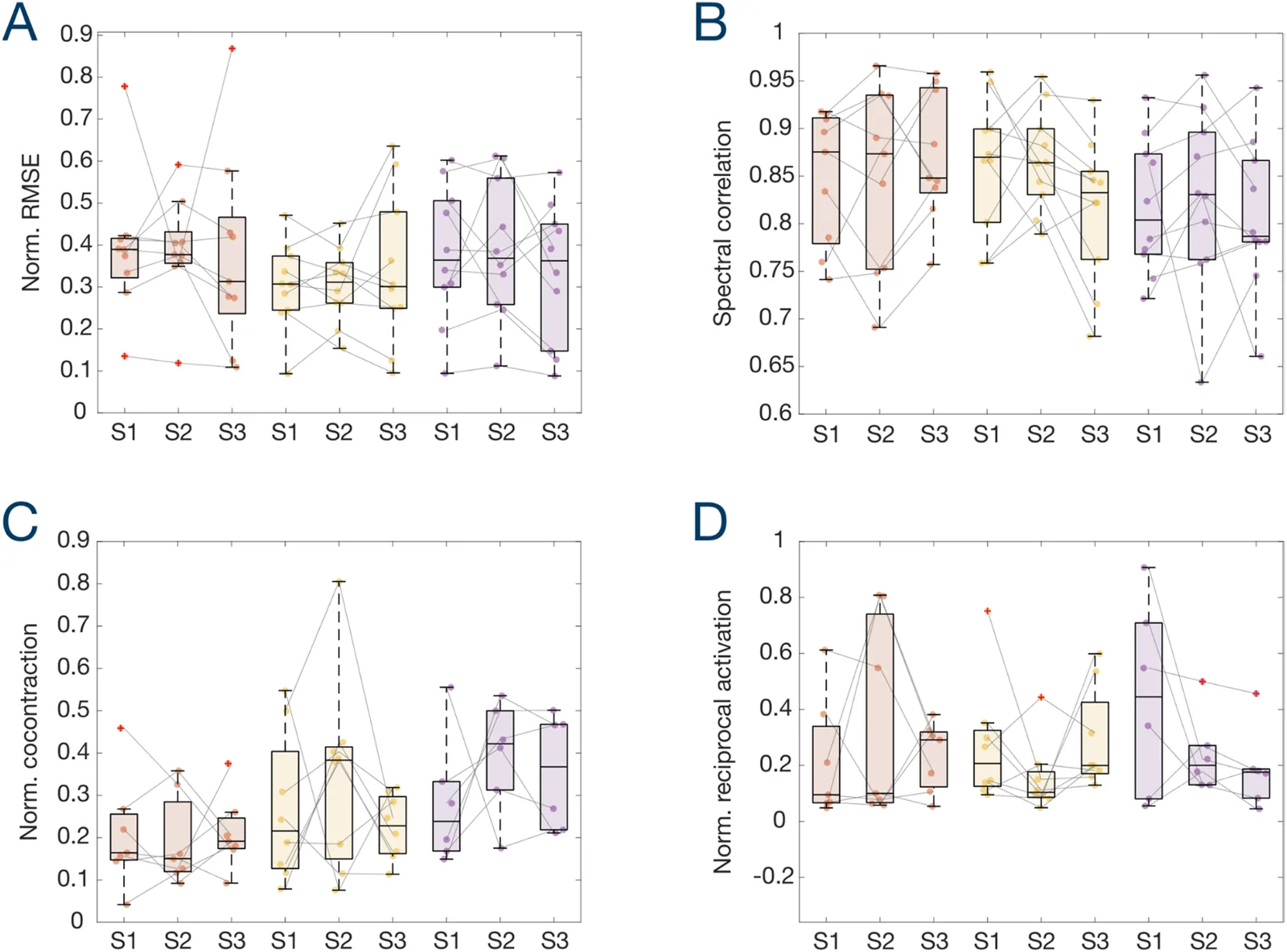
Temporal evolution of the unassisted performance and muscle activity during the target-tracking task (S1: Session 1, S2: Session 2, S3: Session 3). The plots in the red colour present data from patients in the FES group, while the yellow represents patient in the Robot group, and purple in the Hybrid group. The grey lines are used to connect data points from the same patient over the different sessions. A. Effect of the training on the normalised RMSE between the patient and target trajectory. B. Effect of the training on the correlation between the frequency spectra of the patient and target trajectory. C. Effect of the training on the cocontraction of the wrist flexor and extensor muscles. D. Effect of the training on the reciprocal activation of the wrist flexor and extensor muscles.

For the normalised RMSE between the patient and the target trajectories (Figure 4A), no significant main effect of Session (*F*(1,134.1) = 0.35, *p* = 0.55) or Session × Group interaction (*F*(1,132.8) = 0.066, *p* = 0.063) was found, indicating no systematic improvement in unassisted performance across sessions.

Similarly for the spectral correlation (Figure 4B), there was no significant main effect of Session (*F*(1,134.3) = 1.24, *p* = 0.27) or Session × Group interaction (*F*(1,134.5) = 1.62, *p* = 0.20), indicating that the tracking performance did not improve consistently across repeated sessions. This suggests that the main gains in performance occur during assisted trials within sessions, rather than cumulatively across repeated exposure. The expected training or learning effect of the task through this repeated assistive training was not observed here in any of the groups. While some patients’ performances increased over time, the performance of others worsened, suggesting heterogeneous improvement patterns, although patients’ FM-UE scores did not have a significant interaction effect with Session (*p* = 0.949) (Supplemental File 1, Figure 7B).

Regarding cocontraction, a significant Phase × Session × Group interaction was detected (*F*(2,118.6) = 5.17, *p* = 0.0432). However, further exploration of group-wise trends within phases indicated that in the unassisted condition, session trends were small and non-significant across groups (all *p* > 0.09) (Figure 4C). As with the performance metrics, these results suggest that training did not systematically reduce CC within groups over the three sessions and that it is likely that learning did not occur here. Additionally, there was a significant interaction between Session and FM-UE score (*F*(1,118) = 5.17, *p* = 0.025). Post-hoc analyses revealed that session-related trends differed by impairment severity (Supplemental File 1, Figure 7C): mildly impaired patients (FM-UE > 40)^27^ showed a significant reduction in CC across sessions (slope = −0.115 ± 0.055, *p* = 0.0367), whereas a decreasing trend was observed in moderate or severely impaired groups (*p* > 0.057). Pairwise comparisons indicated that session slopes differed significantly between mild and severe patients (*p* = 0.037).

For reciprocal activation, the model revealed significant main effects of Session (*F*(1,123.2) = 4.85, *p* = 0.030) and a Session × Group (*F*(2,122.8) = 8.05, *p* = 0.0005) interaction. However, the interaction between Phase and Session was not significant (*p* = 0.500) (Figure 4D). Group-wise analyses indicated that RA increased significantly over sessions in the Hybrid group (sessions slope = 0.118 ± 0.028, *p* < 0.001), whereas FES and Robot groups showed no significant trends (*p* > 0.3). Similarly, as for the cocontraction, we found a significant interaction between Session and FM-UE score (*F*(1,118) = 12.76, *p* = 0.0005). When analysing session-related trends by impairment severity (Supplemental File 1, Figure 7D), we also observed a significant reduction in RA over sessions in mildly impaired patients (slope = −0.0773 ± 0.0335, *p* = 0.0225), but no changes in the moderate and severely impaired groups (*p* > 0.1). Pairwise comparisons between the severity groups indicated that session slopes differed significantly between all severity groups (all *p* = 0.0015).

## Discussion

This study investigated the feasibility of using a simple hybrid robot-FES system in a clinical environment with subacute stroke patients. The goal here was to characterise the impact of robotic, FES, and combined hybrid assistance on user experience, motor performance, effort, fatigue, and to guide the development of future patient-centred hybrid control strategies.

User experience is consistently positive for the robot, FES and combined hybrid modalities. Patients across all groups rated the system as comfortable, enjoyable to use, and desirable for continued use, with hybrid assistance showing slightly more clustered positive responses (Figure 2A). Most studies did not consider subjective assessment of the assistance as part of their hybrid system evaluation, but a few investigated its usability.^15,28^ In a study combining electrical stimulation with passive robotic support, participants reported that the stimulation was comfortable and the training enjoyable.^28^ In contrast, another study involving a passive exoskeleton with FES assistance found only moderate usability.^15^ Our qualitative findings complement the existing literature and provide preliminary evidence that the proposed system is well tolerated and engaging in a clinical environment. In this context, our qualitative findings extend existing evidence by showing that the proposed system is both well tolerated in the clinic and perceived as engaging by stroke patients.

Interestingly, although patients consistently perceived assistance across all modalities, their ratings of its usefulness were mixed (Figure 2B and C). This suggests that the assistance did not consistently align with participants’ intended movements, as control was driven by the target trajectory rather than their voluntary effort. Similar findings have been reported in robotic rehabilitation, where patients described the device as opposing their movements.^29^ In the present study, the variability was most evident in the Robot group, likely due to the relatively low torque levels chosen to avoid slacking, which may have rendered the assistance less perceptible or less effective. In contrast, electrical stimulation was more noticeable and often rated as useful, despite its less consistent influence on performance metrics. Moreover, the assistance in the Hybrid group was the combined assistance from the FES and the Robot group, hence explaining a consistently higher rating across participants.

Immediate assistance improves tracking error in the time-domain but not consistently in the frequency-domain. The results from the mixed-effects analyses in this study indicate that, in general, assistance improved tracking error compared to unassisted trials, irrespectively of the assistance modalities (Figure 3A and B). Within the present study, Hybrid assistance was not associated with significantly greater gains in tracking performance even though patients received the addition of robotic assistance (as patients in the Robot group) and FES assistance (as patients in the FES group), and it did not outperform the Robot assistance. This is in line with our observations in unimpaired participants^20^ and results from other studies.^30,31^ These findings suggest that FES alone can improve tracking performance during the task, which is in agreement with previous work in the literature indicating that at a slow target speed (10∘/*s*), there is no significant difference in RMSE with various combinations of robot and FES assistance applied to wrist flexion/extension.^32^

Furthermore, the interaction between Phase and FM-UE was significant, indicating that patients with greater impairment showed larger reductions in RMSE with assistance. This may be due to the fixed amount of assistance provided to all patients, which may have led to a ceiling effect in less impaired participants and highlights the potential benefit of personalised assist-as-needed control strategies. On the other hand, different results were obtained with the spectral correlation where we did not see a significant global Phase effect, although a significant FM-UE × Phase interaction was found, similarly as in the RMSE. Although there was no interaction with Group, the grey lines in Figure 3A and B suggest that patients in the FES group had a lower RMSE with assistance, while this trend is not clear for the spectral correlation. Instead, all patients in the Robot group improved in both metrics.

This suggests that stimulation can reduce positional error but is less effective in shaping temporal accuracy This is likely due to the electromechanical delay, i.e. the delay between the stimulation onset and the induced motion.^4^ The variability in individual performance improvements may reflect a more heterogeneous response to stimulation compared to the robotic assistance. To further investigate this trend, the Spearman’s correlation between the FM-UE score and the assistance effect (difference in spectral correlation between the unassisted and assisted trials) was computed (Supplemental File 1, Figure 6B). We found negative correlations for patients in the Robot (*ρ* = −0.22) and Hybrid (*ρ* = −0.51) groups, indicating that patients with stronger impairment (lower FM-UE) benefitted more from the assistance in terms of spectral correlation, which was also observed in the LME results. However, in the FES group, this correlation is positive (*ρ* = 0.20), hence showing that patients with low motion abilities did not necessarily improve their ability to track the target’s frequency pattern with FES assistance. This provides further evidence that FES may not be useful in improving accuracy in the frequency domain. In addition to the electromechanical delay, this may also be due in part to the fact that the comfortable stimulation amplitude range in some patients was limited and could not generate strong torque, although it could contract muscles. Muscle activity patterns reveal differences in recruitment strategies between the assistance modalities. We found that cocontraction levels increased significantly from the unassisted to the assisted trials for patients in the FES group, but there were no significant changes in the Robot group and a slight trend for patients in the Hybrid group. The elevated muscle activity when stimulation is applied is expected as this adds to the natural contraction induced during volitional activation in the unassisted trials. Such observations are consistent with our study in unimpaired participants^20^ and with the literature.^32,33^ The larger cocontraction in the FES and Hybrid groups may also indicate some level of counteraction between the user’s intention and the delayed stimulation, or between the robotic torque and the torque induced by the FES, delayed with respect to the robotic assistance. The interaction between the FM-UE score and the Phase further indicated that patients with higher impairments tended to show a larger reduction in cocontraction in the assisted phase. This may be in part due to the electromechanical delay where patients who are able to perform the task without assistance may find the FES assistance counteracting their motion. This could also be associated with a reduced volitional effort in patients with severe impairments once this assistance is provided. In addition, we did not find significant signs of muscle fatigue as observed when investigating the change in several frequency-based EMG metrics (Supplementary File 2), suggesting that there was no stronger increase in muscular fatigue in the FES group compared to the Robot group as we had expected. This is also observed in the patients’ perception of the mental and physical demand of the task, where the median answers across the three intervention groups were below 5/10 and no difference were found between groups.

*Training over three sessions did not induce consistent improvements in unassisted tracking performance and physiological measures.* Although task-related improvements were expected in this study due to the training effect over the three sessions, there were no significant changes in unassisted performance over time (Supplemental File 1, Figure 6A and B). Similarly, group-wise reductions in cocontraction during the unassisted trials, which typically indicate motor learning, were not significant, although a significant interaction between Phase, Session and Group was found. This supports the observation from the tracking metrics. This is likely due to the short intervention periods, where three days spread across a week are not sufficient to drive measurable motor learning, even though only improvements in the trained sensorimotor task were expected.

Furthermore, we found that the FM-UE scores had an effect on the changes in cocontraction and reciprocal activation over sessions, where mildly impaired patients (FM-UE > 40) showed significant reductions in physiological measures, indicating learning, while this was not the case in the more impaired patients (Supplemental File 1, Figure 7C and D). This may reflect that patients with milder impairments could already benefit from this short training while patients with lower motor capabilities need longer training before physiological adaptations can be observed. This may also be related to the nature of the task, requiring continuous motor control and hand-eye coordination, and which may be too advanced for patients with more severe impairments. However, the significant interaction between FM-UE and assistance indicates that patients across a wide range of impairment severities were able to benefit from the immediate assistance during the task. Indeed, patients with very severe upper-limb impairments, including eight patients with FM-UE scores lower than ten could participate in the study and could improve their tracking performance with the assistance, while enjoying the training These findings suggest that the proposed system and protocol can be applied across a broad range of impairment severities, even using a standardised assistance level. Moreover, the FM-UE scores were balanced across groups but differences in baseline performance and EMG levels suggest potential group-specific biases and participants may benefit from stronger assistance or a more challenging task to drive their individual improvements, specifically those with more severe impairments. This approach would however reduce the standardisation of the protocol, potentially complicating the interpretation of the results, especially when the primary aim is not to conclude on the clinical efficacy of an intervention.

*This study has several limitations that should be considered when interpreting the findings.* First, the sample size within each intervention group was modest, limiting statistical power, particularly for between-group comparisons and longitudinal analyses. Second, the intervention consisted of only three training sessions over approximately one week, which was primarily designed to evaluate feasibility and short-term responses rather than clinical efficacy or motor recovery. Consequently, the absence of significant training effects should not be interpreted as evidence that longer-term hybrid training is ineffective. Third, participants presented with a broad range of impairment severities, although baseline FM-UE scores did not differ significantly between groups. This clinical heterogeneity likely contributed to the variability observed in performance and EMG outcomes. Finally, the assistance parameters were intentionally standardised across participants to allow comparison between intervention modalities. While this improved experimental consistency, it may not have provided an optimal challenge for every participant and may have limited the ability to detect differential effects of the three assistance strategies. Accordingly, the present findings should be interpreted primarily as evidence supporting the feasibility, acceptability, and immediate assistive effects of the proposed system rather than definitive evidence of clinical superiority of one modality over another.

## Conclusions

This study evaluated the feasibility and short-term effects of robotic, FES, and hybrid assistance during a wrist-tracking task in subacute stroke patients undergoing inpatient rehabilitation. All assistive modalities were found acceptable, enjoyable, and useful, with hybrid assistance receiving the most consistent positive feedback. Assistance reduced tracking error during the task across all modalities, demonstrating immediate assistive effects under the experimental conditions, although no significant differences between intervention groups were observed. Muscle activity patterns showed modality-specific recruitment, with stimulation promoting active engagement, though it may have been associated with antagonist activation. Even patients with severe impairments benefited from assistance and could engage with the training, suggesting that the system can be used across a broad range of impairment severities, although confirmation in larger studies is needed. The short training duration and uniform task parameters limited the detection of learning effects and constrained therapy individualisation. Training-related adaptations were observed mainly in mildly impaired patients, who showed reduced cocontraction and reciprocal activation, while more severely impaired patients may require longer exposure. Future studies with larger cohorts and longer intervention periods are required to determine the clinical efficacy of hybrid robot-FES rehabilitation and to evaluate whether personalized assistance strategies provide additional benefits.

## Supplemental material

Supplemental material - Hybrid robot & FES rehabilitation after stroke: Short-term performance, fatigue, and patient experience Supplemental material for Hybrid robot & FES rehabilitation after stroke: Short-term performance, fatigue, and patient experience by Lucille Cazenave, Chiara Höhler, Satoshi Endo, Hossein Kavianirad, Nuria Peña-Pérez, Lisa-Marie Huber, Aaron Yurkewich, Thierry Keller, Sandra Hirche, Klaus Jahn, Etienne Burdet, and Carmen Krewer in Journal of Rehabilitation and Assistive Technologies Engineering.

Supplemental material - Hybrid robot & FES rehabilitation after stroke: Short-term performance, fatigue, and patient experience Supplemental material for Hybrid robot & FES rehabilitation after stroke: Short-term performance, fatigue, and patient experience by Lucille Cazenave, Chiara Höhler, Satoshi Endo, Hossein Kavianirad, Nuria Peña-Pérez, Lisa-Marie Huber, Aaron Yurkewich, Thierry Keller, Sandra Hirche, Klaus Jahn, Etienne Burdet, and Carmen Krewer in Journal of Rehabilitation and Assistive Technologies Engineering.

## Data Availability

The datasets collected during the current study are not publicly available due to patient privacy and institutional ethical restrictions.*
