# Supplementary material for: Hybrid robot & FES rehabilitation after stroke: Short‐term performance, fatigue, and patient experience
Source: J Rehabil Assist Technol Eng. 2026 Sep 23;13:20556683261488467. doi: 10.1177/20556683261488467 (PMC13760121; doi:10.1177/20556683261488467)
Supplement: Supplementary file 1 [file sj-pdf-1-jrt-10.1177_20556683261488467.pdf]

Hybrid Robot & FES Rehabilitation after Stroke: Short-Term Performance, Fatigue, and Patient Experience

Supplementary File 1 – Additional Figures

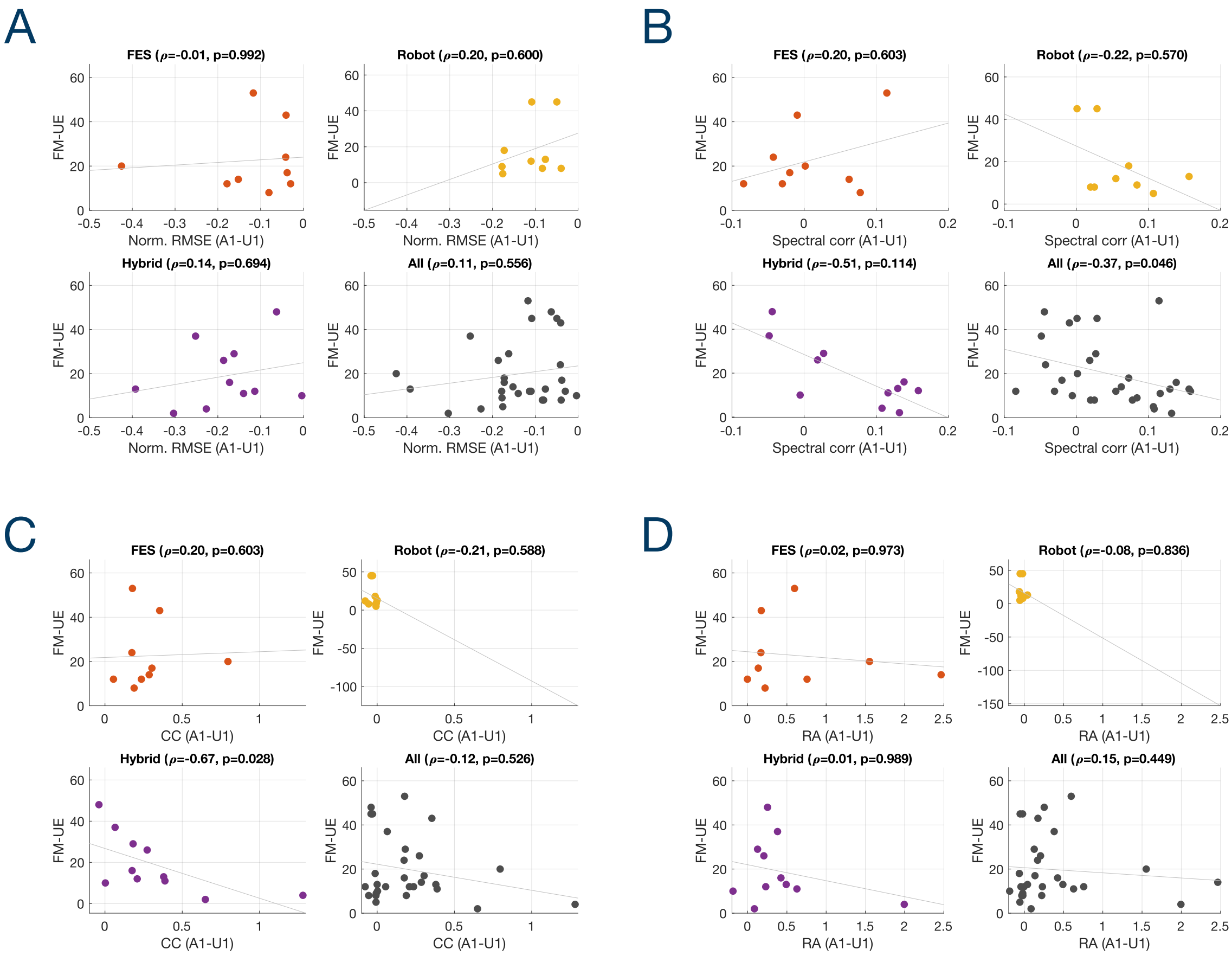

Figure 6. Spearman's correlation between FM-UE scores and assistance effect (assisted performance – unassisted performance) on various metrics: normalised RMSE **(A)**, spectral correlation **(B)**, cocontraction **(C)**, and reciprocal activation **(D)**, for each group individually and the whole population. A performance improvement is indicated by a negative RMSE difference and a positive spectral correlation difference. A negative cocontraction or reciprocal activation indicates a reduction in physiological measures with assistance.

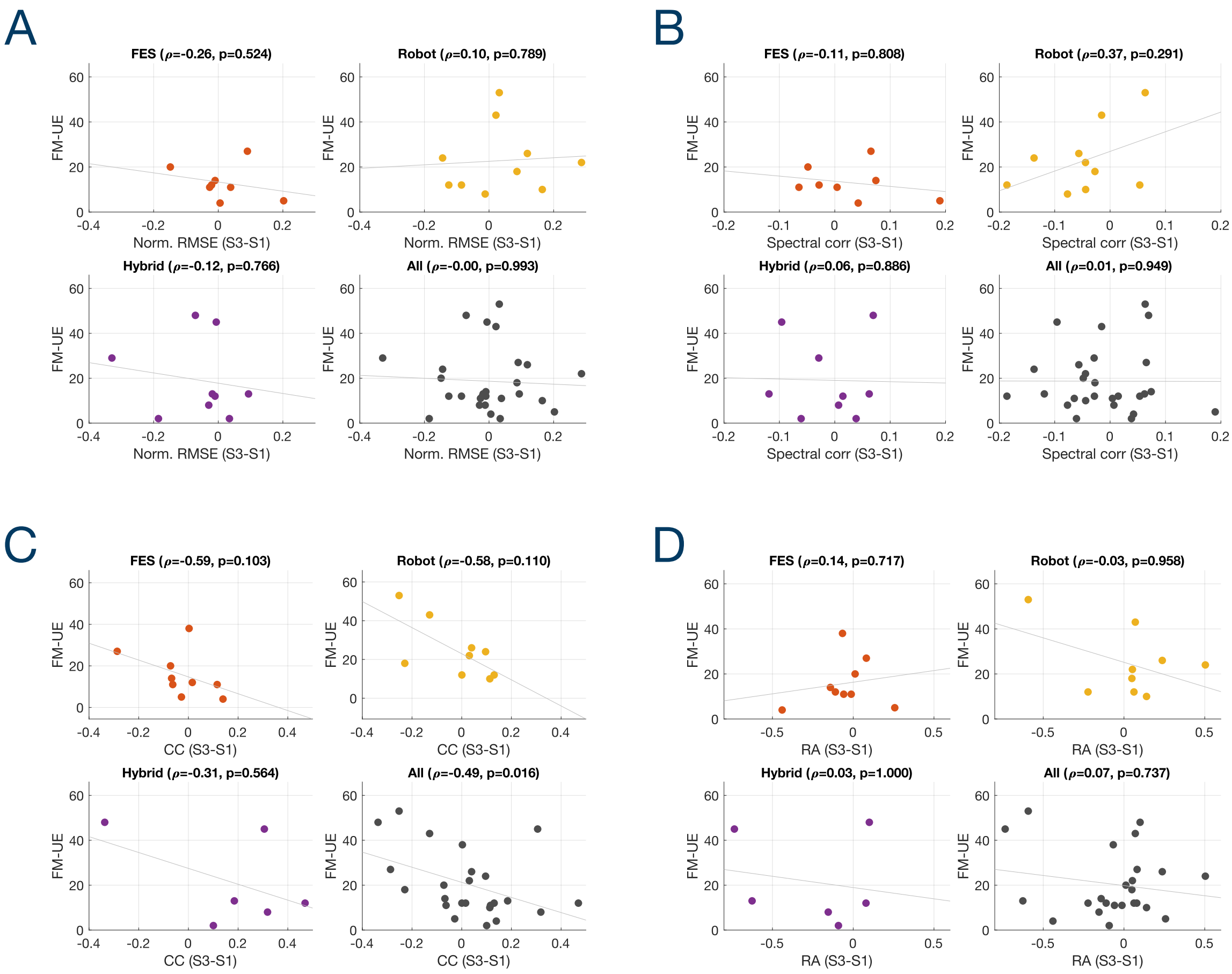

Figure 7. Spearman's correlation between FM-UE scores and improvement in unassisted trials over sessions (S3 – S1) on various metrics: normalised RMSE **(A)**, spectral correlation **(B)**, cocontraction **(C)**, and reciprocal activation **(D)**, for each group individually and the whole population. A performance improvement is indicated by a negative RMSE difference and a positive spectral correlation difference. A negative cocontraction or reciprocal activation indicates a reduction in physiological measures from Session 1 to 3.
