# Supplementary material for: Hybrid robot & FES rehabilitation after stroke: Short‐term performance, fatigue, and patient experience
Source: J Rehabil Assist Technol Eng. 2026 Sep 23;13:20556683261488467. doi: 10.1177/20556683261488467 (PMC13760121; doi:10.1177/20556683261488467)
Supplement: Supplementary file 2 [file sj-pdf-2-jrt-10.1177_20556683261488467.pdf]

Hybrid Robot & FES Rehabilitation after Stroke: Short-Term Performance, Fatigue, and Patient Experience

Supplementary File 2 – Fatigue analysis

Computation of fatigue metrics

Fatigue metrics were derived from the pre-processed surface EMG recordings, when these were available (EMG was not recorded for ten patient-session pairs due to technical issues). EMG pre-processing consisted of Notch filtering at the mains noise frequency of 50 Hz, followed by high-pass and low-pass second order Butterworth filters (with cut-off frequencies f_c = 10 Hz and f_c = 500 Hz, respectively).

To evaluate whether the training induces muscular fatigue, the change in frequency-domain EMG metrics before and after the training was computed, using the muscle activity signals recorded in the unassisted trials. The *median frequency* of the filtered EMG signal was obtained from its Fourier transform, where a decrease typically corresponds to fatigue[1,2]. The *Choi-Williams time-frequency distribution*[3] was also used to extract the instantaneous median frequency of the time-dependent power spectrum, and using the mean and variance, which can characterise fatigue specifically during dynamic contractions[4–6]. In addition, the *Dimitrov Spectral Index*, which is the ratio of the signal spectral moment of order (-1) and the spectral moment of order five was computed as it has also been found relevant for detecting muscle fatigue[7]. The difference in these metrics in the unassisted trials preceding and following the training block was computed.

Statistical analysis

To investigate the potential effect of the intervention group on the difference in frequency-domain EMG metrics, used to characterise fatigue, we conducted a one-way ANOVA or a

Kruskal-Wallis test, if the normality assumption was not satisfied. No post-hoc tests were applied as the omnibus test was not significant.

All analyses followed an intention-to-treat principle, whereby all available data were included even when some sessions were missing, except for longitudinal (3 x 3) analyses, which required complete data across all sessions. A significance level of 0.05 was applied to all statistical tests.

Fatigue results

We investigated whether changes in EMG metrics in the frequency domain, typically used to characterise fatigue, were observed after patients completed the assisted training block (Fig. 8). Across all metrics, i.e. median frequency (A, B), mean of the instantaneous median frequency (C, D) and Dimitrov spectral index (E, F), no statistically significant differences were found between intervention groups (all $p > 0.1$). This was consistent for both flexor and extensor muscles. A negative difference would indicate a decrease in the metric value after the assisted training block, suggesting a rise in muscle fatigue for the median frequency (Fig. 8 A, B) and the instantaneous median frequency (Fig. 8 C, D), while a positive difference, i.e. an increase in the Dimitrov spectral index would indicate fatigue (Fig. 8 E, F). Here, most of the median frequency metrics in the three intervention groups are above zero indicating no sign of fatigue (Fig. 8 A-D), except in the Robot group where we can observe a negative median in the median frequency and mean of the instantaneous median frequency of the extensor EMG (Fig. 8 B, D), although this is not significantly different from zero (both $p > 0.4$). Similarly for the Dimitrov spectral index, where the median for the Robot group is slightly above zero in the flexor and extensor EMG signals, although not significant, hence suggesting that fatigue is not occurring.

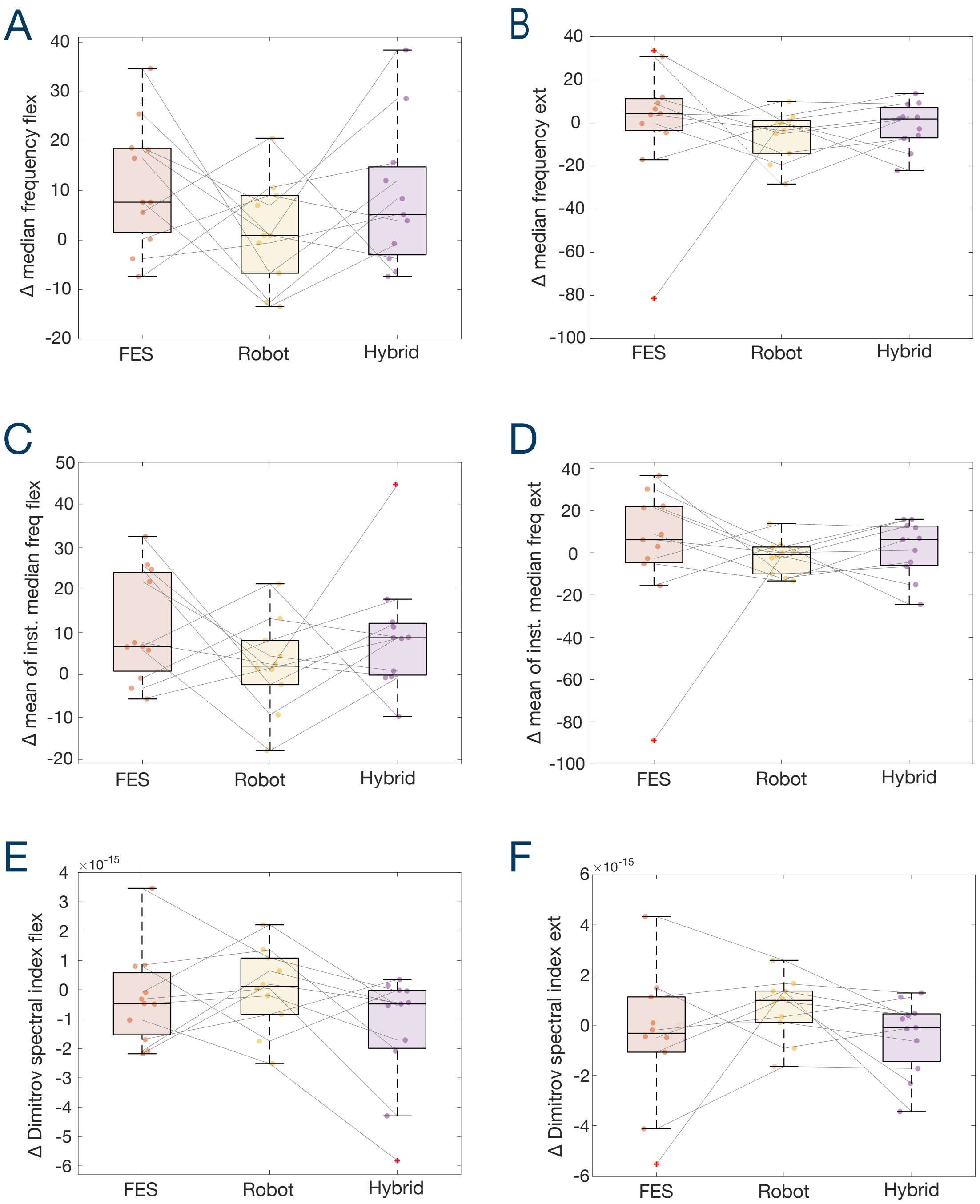

Figure 8. Difference in frequency-based EMG metrics between the last three and first three trials for each condition for the wrist flexor (A, C, E) and the extensor (B, D, F) muscles: the difference in median frequency (A,B) and in the mean of the instantaneous median frequency (C,D), as well as the Dimitrov index (E,F) are presented. A negative difference in the median frequency (A,B) and in the mean of the instantaneous median frequency (C,D) would indicate fatigue. A positive difference in Dimitrov index (E,F) would indicate fatigue. The grey lines are used to connect data points from the same patient under different conditions.

Results interpretation and discussion

When assisted by a robot and/or FES during a target-tracking task, patients did not feel tired, and no significant sign of muscle fatigue was observed. The rise of fatigue during the task was investigated by comparing several frequency-based EMG metrics, including those suited for a dynamic task[4–6], in the unassisted trials before and after the assisted training (Fig. 8). No significant between-group differences in the change from pre- to post-training were found in any of these metrics, suggesting that there was no stronger increase in muscular fatigue in the FES group compared to the Robot group as we had expected. Furthermore, the medians of the difference in these fatigue indices were mostly close to zero and further statistical investigations did not indicate any significant changes. Hence even when investigating changes within each condition, we did not find any sign of fatigue that would have risen after the assisted training. This corresponds to the subjective answers to the NASA-TLX questionnaire on the perceived mental and physical demands, where the median answers across the three intervention groups were below 5/10 and no difference were found between groups, although one could expect the robot-assisted training to be less demanding or tiring as muscles are not being electrically stimulated. This indicates that under the present task parameters, neither subjective workload nor spectral EMG features indicated an accumulation of muscular fatigue.

Although there is a consensus on the rapid increase in muscle fatigue after electrical stimulation in the literature, this is usually tested with fatiguing protocols in unimpaired cohorts typically involving more intense conditions, such as 1-5 minutes of maximum voluntary contraction or 1-3 minutes of tetanic stimulations[8] or evaluating the effect of different stimulation parameters during sustained stimulation on generated torque to understand the impact on fatigue[9,10]. Fatigue protocols in clinical populations, mostly in individuals with spinal cord injuries, who typically do not have residual voluntary

contraction, and also follow similar procedures[11–13], which do not necessarily resemble the motor training tasks for stroke patients. This makes their results less applicable to a typical rehabilitation scenario. A repetitive FES-assisted grasping protocol, which can also be assimilated to a rehabilitation session, found that muscle fatigue did not increase over time[14].

References

1. Stulen FB, De Luca CJ. Frequency Parameters of the Myoelectric Signal as a Measure of Muscle Conduction Velocity. *IEEE Trans Biomed Eng*. 1981;BME-28(7):515-523. doi:10.1109/TBME.1981.324738

2. Arendt-Nielsen L, Mills KR, Forster A. Changes in muscle fiber conduction velocity, mean power frequency, and mean EMG voltage during prolonged submaximal contractions. *Muscle Nerve*. 1989;12(6):493-497. doi:10.1002/mus.880120610

3. Choi HI, Williams WJ. Improved time-frequency representation of multicomponent signals using exponential kernels. *IEEE Trans Acoust Speech Signal Process*. 1989;37(6):862-871. doi:10.1109/ASSP.1989.28057

4. Karlsson S, Jun Yu, Akay M. Time-frequency analysis of myoelectric signals during dynamic contractions: a comparative study. *IEEE Trans Biomed Eng*. 2000;47(2):228-238. doi:10.1109/10.821766

5. Bonato P, Roy SH, Knaflitz M, De Luca CJ. Time-frequency parameters of the surface myoelectric signal for assessing muscle fatigue during cyclic dynamic contractions. *IEEE Trans Biomed Eng*. 2001;48(7):745-753. doi:10.1109/10.930899

6. González-Izal M, Malanda A, Navarro-Amézqueta I, et al. EMG spectral indices and muscle power fatigue during dynamic contractions. *J Electromyogr Kinesiol*. 2010;20(2):233-240. doi:10.1016/j.jelekin.2009.03.011

7. Dimitrov GV, Arabadzhiev TI, Mileva KN, Bowtell JL, Crichton N, Dimitrova NA. Muscle Fatigue during Dynamic Contractions Assessed by New Spectral Indices. *Med Sci Sports Exerc*. 2006;38(11):1971-1979. doi:10.1249/01.mss.0000233794.31659.6d

8. Rodriguez-Falces J, Place N. Determinants, analysis and interpretation of the muscle compound action potential (M wave) in humans: implications for the study of muscle fatigue. *Eur J Appl Physiol*. 2018;118(3):501-521. doi:10.1007/s00421-017-3788-5

9. de Kroon J, IJzerman M, Chae J, Lankhorst G, Zilvold G. Relation between stimulation characteristics and clinical outcome of the upper extremity in stroke. *J Rehabil Med Off J UEMS Eur Board Phys Rehabil Med*. 2005;37:65-74. doi:10.1080/16501970410024190

10. Dreibati B, Lavet C, Pinti A, Poumarat G. Influence of electrical stimulation frequency on skeletal muscle force and fatigue. *Ann Phys Rehabil Med*. 2010;53(4):266-277. doi:10.1016/j.rehab.2010.03.004

11. Heasman JM, Scott TRD, Vare VA, et al. Detection of fatigue in the isometric electrical activation of paralyzed hand muscles of persons with tetraplegia. *IEEE Trans Rehabil Eng*. 2000;8(3):286-296. doi:10.1109/86.867870

12. Estigoni E, Fornusek C, Hamzaid N, Hasnan N, Smith R, Davis G. Evoked EMG versus Muscle Torque during Fatiguing Functional Electrical Stimulation-Evoked Muscle Contractions and Short-Term Recovery in Individuals with Spinal Cord Injury. *Sensors*. 2014;14(12):22907-22920. doi:10.3390/s141222907

13. Qiu S, Draghici AE, Picard G, Taylor JA. Muscle Fatigue in Response to Electrical Stimulation Pattern and Frequency in Spinal Cord Injury. *PM&R*. 2020;12(7):699-705. doi:10.1002/pmrj.12282

14. Höhler C, Endo S, Hermsdörfer J, et al. Repetitive Grasping After Stroke Assisted by Functional Electrical Stimulation. *Artif Organs*. Published online June 27, 2025:aor.15033. doi:10.1111/aor.15033
